# Large Recovery of Fish Biomass in a No-Take Marine Reserve

**DOI:** 10.1371/journal.pone.0023601

**Published:** 2011-08-12

**Authors:** Octavio Aburto-Oropeza, Brad Erisman, Grantly R. Galland, Ismael Mascareñas-Osorio, Enric Sala, Exequiel Ezcurra

**Affiliations:** 1 Marine Biology Research Division, Scripps Institution of Oceanography, La Jolla, California, United States of America; 2 Centro para la Biodiversidad Marina y la Conservación, La Paz, BCS, Mexico; 3 National Geographic Society, Washington, DC, United States of America; 4 Centre d'Estudis Avançats de Blanes (CSIC), Blanes, Spain; 5 UC-MEXUS, University of California Riverside, Riverside, California, United States of America; Institute of Marine Research, Norway

## Abstract

No-take marine reserves are effective management tools used to restore fish biomass and community structure in areas depleted by overfishing. Cabo Pulmo National Park (CPNP) was created in 1995 and is the only well enforced no-take area in the Gulf of California, Mexico, mostly because of widespread support from the local community. In 1999, four years after the establishment of the reserve, there were no significant differences in fish biomass between CPNP (0.75 t ha^−1^ on average) and other marine protected areas or open access areas in the Gulf of California. By 2009, total fish biomass at CPNP had increased to 4.24 t ha^−1^ (absolute biomass increase of 3.49 t ha^−1^, or 463%), and the biomass of top predators and carnivores increased by 11 and 4 times, respectively. However, fish biomass did not change significantly in other marine protected areas or open access areas over the same time period. The absolute increase in fish biomass at CPNP within a decade is the largest measured in a marine reserve worldwide, and it is likely due to a combination of social (strong community leadership, social cohesion, effective enforcement) and ecological factors. The recovery of fish biomass inside CPNP has resulted in significant economic benefits, indicating that community-managed marine reserves are a viable solution to unsustainable coastal development and fisheries collapse in the Gulf of California and elsewhere.

## Introduction

Overfishing has impacted marine biodiversity and ecosystems both directly (through removal of significant biomass) and indirectly (by changing ecological linkages) throughout history [1], [2]. No-take marine reserves have been proposed as one of the most successful management tools to reverse these degradation trends [e.g., 3]. Evidence supporting the positive effects of no-take reserves include a greater abundance and biomass of fish inside marine reserves than in fished areas [see meta–analysis in 4]; an exponential increase of predatory fish biomass [e.g.], [ 5,6]; and shifts in species composition and trophic cascades that result in the restoration of natural marine communities within protected areas [7]–[10]. While these ecological changes operate on decadal times scales through a series of transient states [5], [11], [12], initial detections of both direct effects of area closures on target species and indirect effects on other taxa through cascading trophic interactions can be observed much sooner (5 and 13 years, respectively) [13], [14].

In addition to the aforementioned conservation benefits, well-enforced marine reserves help reduce local poverty and increase the economic revenue of coastal communities [15], [16]. Protected areas with locally managed resources and stakeholder buy-in can be more successful than areas with top down, federally mandated preservation [see 17]. However, marine reserve agendas have faced considerable opposition from different sectors of the society (e.g. commercial and recreational fisheries), only 0.1 percent of the world's ocean is completely protected from extractive activities, and most reserves suffer from poor management and enforcement [18], [19]. Moreover, the long-term success of marine reserves is a social issue that requires strong local leadership, social cohesion, involvement and effective self-enforcement within the community, and inter-generational coordination [20], [21].

Most of our knowledge of the benefits produced by no-take marine reserves comes from reserves smaller than 10 km^2^, and from single-time comparisons between protected areas and nearby fished sites [e.g.], [ 4], [13], [22,23]. Large relative increases in fish biomass (up to 20-fold) have been observed [e.g., 5]; but since these recoveries have happened in reserves less than 1 km^2^, the absolute increase in biomass has been limited. Furthermore, few marine reserves have been able to restore fish biomass to values similar to unfished habitats [24], [25]. Maybe because of that, most ecological and economic benefits (via spillover of adults to nearby unprotected areas) have been found for distances of only one kilometer on average beyond the reserve's boundaries [26].

Fisheries regulations in the Gulf of California (GOC) are numerous and complex as a result of the large number of exploited marine resources, and they have not been successful in recovering fish stocks [27], [28]. Even with its relatively low population size, the GOC is no exception to worldwide coastal and marine degradation trends. Overfishing, destruction of critical habitats, and the lack of proper planning that outline conservation and fisheries management priorities threaten marine biodiversity in the GOC [e.g.], [ 28]–[32]. Large scale tourism developments in some areas in the GOC, though often touted as an alternative to fishing, have exacerbated problems by increasing local population sizes, fishing effort to feed tourists, and human-nature interactions [33]. These issues will become more severe as commercial and transportation development continues to grow in the region [34], but while efforts to restore degraded ecosystems (e.g. mangrove forests) are improving, no studies have demonstrated that recovery of GOC marine ecosystems is plausible.

No-take marine reserves currently represent the most widely-promoted tool for the conservation and restoration of coastal and marine ecosystems in the GOC [35], especially in the absence of strong governmental enforcement programs at both national and regional scales. However, most Marine Protected Areas (MPAs) in the GOC, are multiple use areas that include zones ranging from open access to no-take areas; although no-take areas represent less than 5% of the majority of MPAs [36], [37], and have not recorded any significant positive changes in terms of recovery of fish or economic benefits [17].

**Figure 1:**
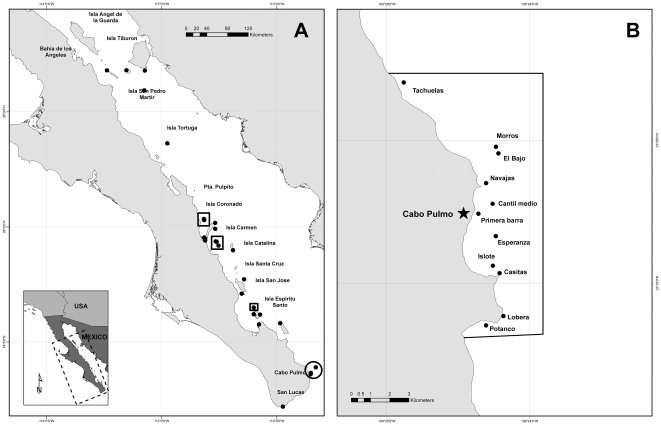
Location of sampling sites. A) Sites surveyed by Sala et al. in 1999 [35], and resurveyed here in 2009. Dots inside the circle represent sites surveyed in Cabo Pulmo National Park in 1999. Dots inside squares represent sites in core zones (no-take areas) of other marine protected areas; the rest represent open access sites. B) Map of 11 sites surveyed at Cabo Pulmo National Park in 2009.

An outlier among GOC MPAs is Cabo Pulmo National Park (CPNP), an area near the southern end of the Baja California Peninsula, designated a National Park in 1995 mainly to protect its large coral communities [38]. CPNP, though one of the smallest MPAs in the region (Table 1), has the largest percentage of core (no-take) area (35%). Due to the determined action of local families, protection and enforcement as a no-take reserve has expanded to include nearly 100% of CPNP's area. In 1999, we visited 60 reefs throughout the GOC from Cabo San Lucas at the southern tip of the Baja California Peninsula to the Midriff Islands in the Upper GOC [35], including reefs inside CPNP (Fig. 1). We replicated that study ten years later. Here we report the changes in fish diversity and biomass at CPNP, relative to other MPAs and unprotected areas in the GOC, between 1999 and 2009.

**Table 1:** List of MPAs established by the Mexican Federal Government in the Gulf of California.

Marine Protected AreasGulf of California	Date Established	Marine Area (km^2^)	No-take (km^2^)	No-take %
**Cabo San Lucas** *Flora and Fauna Area Protection	1973	40	0	0
**Alto Golfo de California y Delta del Río Colorado**Biosphere Reserve	1993	5,416	882.5	16.3
**Cabo Pulmo** *National Park	1995	71	25.0	35.1
**Bahía de Loreto** *National Park	1996	1,837	1.3	0.07
**Archipielago Islas Marías**Biosphere Reserve	2000	6,173	148.4	2.4
**Archipiélago de Espiritu Santo** *National Park	2000	487	6.7	1.4
**Isla San Pedro Martir** *Biosphere Reserve	2002	299	11.1	3.7
**Archipiélago San Lorenzo** *National Park	2005	584	88.0	15.1
**Archipiélago Islas Marietas**National Park	2005	14	0.8	5.7
**Bahia de los Angeles, Canal de Ballenas y Salsipuedes** *Biosphere Reserve	2007	3,880	2.1	0.05

*MPAs with rocky reefs included in this study.

## Results

Fish species richness increased significantly at CPNP from 1999 (average = 15 species per transect) to 2009 (25 species per transect; ANOVA, *p*<0.01). In contrast, reefs inside no-take areas (“core zones”) in other MPAs (1999 = 22, 2009 = 18; ANOVA, *p*<0.0001) and in open access areas (1999 = 20, 2009 = 17, ANOVA, *p*<0.0001) showed a significant decrease in species richness. Additionally, the diversity of top predators (measured as the inverse of Simpson's Index) increased significantly between 1999 and 2009 at CPNP (*p*<0.05); while elsewhere in the GOC, it either remained the same or decreased significantly (see Table S1).

**Figure 2:**
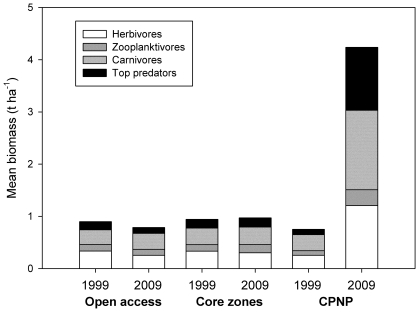
Average biomass of fish trophic groups surveyed in 1999 and 2009 in each site category in the Gulf of California.

In 1999, fish biomass at CPNP was not significantly different from that in the no-take areas or core zones in other MPAs and in open access areas (Fig. 2; Student's *t*-test, *p*>0.05). Between 1999 and 2009 fish biomass increased significantly in all trophic groups at CPNP at annual rates varying between 12 and 25% (Table 2). After 10 years, total biomass at CPNP increased from 0.75 to 4.24 t ha^−1^, a dramatic increase of 3.49 t ha^−1^ that corresponds to a 463% change. Change in fish biomass for each trophic group was higher at CPNP than at other core zones or in open access areas (sign test, *p* = 0.03). Furthermore, while growth rates at CPNP differed significantly from the null hypothesis of zero growth for all trophic groups (*p*<0.03), in all other areas rates did not differ from zero in any trophic group (Table 2). Consequently, fish biomass at CPNP in 2009 was 5.4 times larger than in other core zones and open access areas (Fig. 2). In contrast, differences in fish biomass between other core zones and open access areas were not different in 1999 or 2009 (Fig. 2; Student's *t*-test, *p* = 0.15).

**Table 2:** Changes in fish biomass between 1999 and 2009 in (a) Cabo Pulmo National Park, (b) other no-take or core zones, and (c) open-access areas.

		TopPredators	Carnivores	Zooplanktivores	Herbivores	Total
Cabo Pulmo	*F*	+				
	*t*	+	+	+	+	+
	*U*	+	+		+	+
	ρ	0.25	0.16	0.12	0.16	0.17
Core zones	*F*				+	
	*t*					
	*U*					
	ρ	0.005	0.005	0.03	−0.01	0.003
Open access	*F*					
	*t*					
	*U*					
	ρ	−0.03	0.009	−0.01	−0.03	−0.01

Rows indicate the test used or parameter measured (*F* for Fisher's equality of variances test; *t* for Student's *t*-test for unequal sample sizes and unequal variances; *U* for Mann-Whitney's rank-sum test, and ρ for the rate of change). Plus signs (+) indicate a significant increase in biomass variance (*F*), in mean biomass (*t*), or in ranked biomass (*U*); blank spaces indicate non-significant changes.

Mean biomass within every trophic group increased significantly between 1999 and 2009 at CPNP (Table 2), and the biomass of top predators increased by 1070% (Fig. 2). The relative variance (calculated as the square of the coefficients of variation) in between-transect biomass also increased significantly for top predators (Table 2), implying that spatial aggregation of fish schools increased significantly during the intervening decade.

Differences in size class frequencies indicate that the largest fishes encountered in our surveys were within CPNP, and that there were more individuals in the largest size classes at CPNP than at other reefs in the GOC. Furthermore, for 25 of 88 species encountered in our transects (e.g., *Mycteroperca* spp., *Lutjanus* spp., and *Scarus* spp.), the largest individuals observed in 2009 were at CPNP.

**Figure 3:**
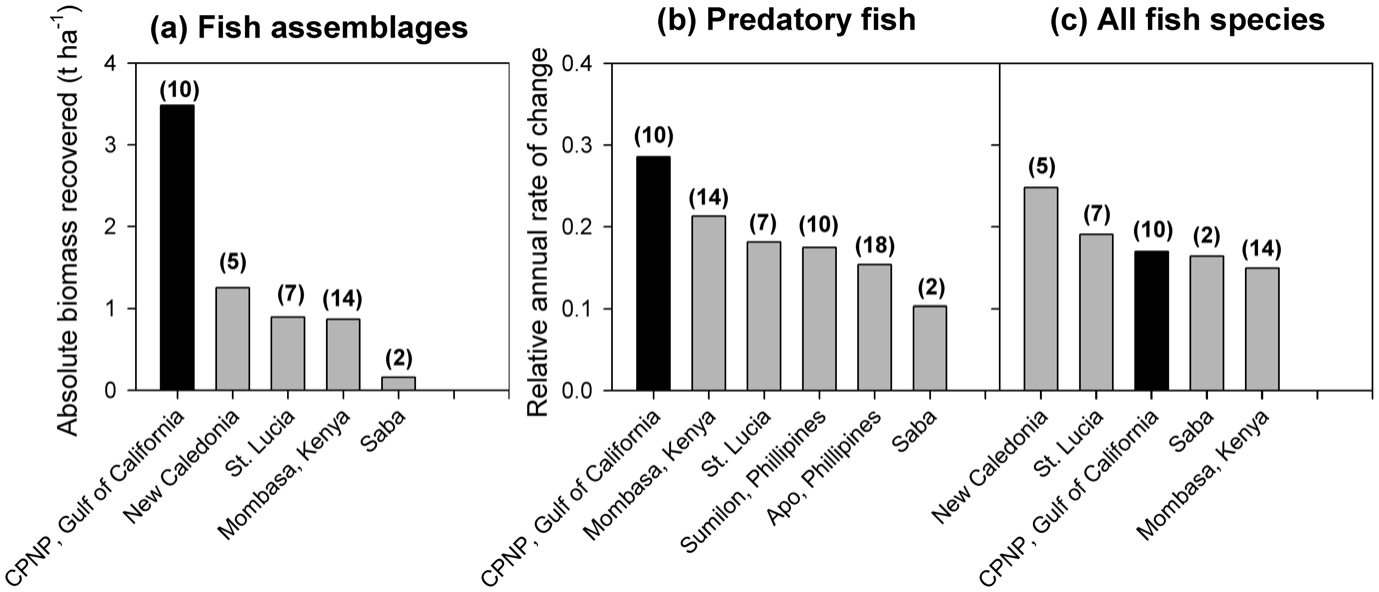
Comparison of the magnitude and rate of change of fish biomass at Cabo Pulmo National Park relative to other marine reserves around the world. Data from other reserves were restricted to the few studies on temporal changes of total fish biomass (including all species) inside the same reserve. In all panels numbers in parentheses above bars represent years between surveys. In panels (b) and (c), relative annual rate of change between time zero and time *t* is calculated as: ρ = ln(*x_t_*/*x*_0_)/*t*. Data sources: Mombasa [6], New Caledonia [7], Saba [43], St. Lucia [45], Sumilon and Apo [44].

CPNP exhibited the largest absolute recovery of biomass in a marine reserve, and the faster relative increase in biomass of top predators, with a 30% annual increase of predatory fish (Fig. 3). To the best of our knowledge, only CPNP and Cabo de Palos Marine Reserve in the Mediterranean [39] have recovered total fish biomass to values larger than 4 t ha^−1^, and shown ratios of biomass inside the reserve to that in the surrounding fished areas larger than 5 times more biomass inside the reserve.

## Discussion

**Figure 4:**
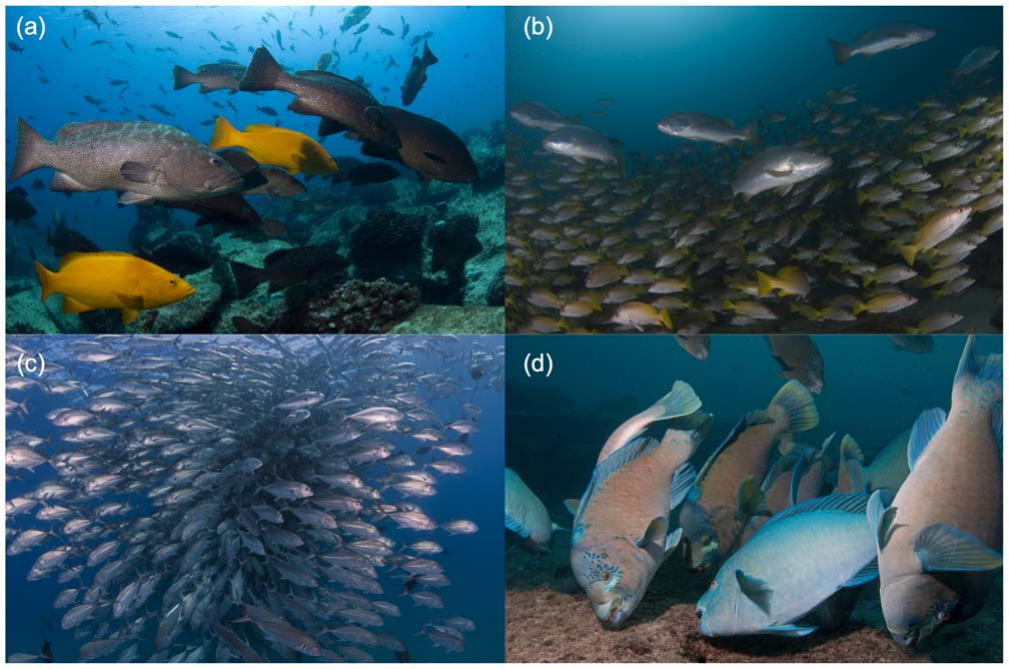
Examples of the fish assemblage at Cabo Pulmo National Park (CPNP). (a) groupers, (b) snappers, (c) jacks, and (d) parrotfishes. Photographs were taken in the summers of 2008–2010 (Photography by: Octavio Aburto).

Our 10-year comparison demonstrates that CPNP has been an effective marine reserve for the recovery of reef fish biomass within its boundaries. After fifteen years of protection, species richness and total biomass are greater, and top predators are more abundant. The larger densities and individual sizes of fish at CPNP (Fig. 4), combine to create an average biomass that is more than five times larger than the average biomass in open access areas in the Gulf of California (GOC).

In contrast to CPNP, core zones in other MPAs in the GOC have not yielded a significant increase in fish biomass or species richness, and are no different from open access areas. For example, two small no-take marine reserves created in 2001 at Loreto Bay National Park [40] have stabilized fish abundances (as opposed to declines observed elsewhere), but probably as a result of their small size (1.4 km^2^ of total no-take area), they have not resulted in the recovery of fish populations [17].

Regrettably, we only sampled two reefs inside CPNP in 1999 (compared to 11 sites in 2009), as we did not anticipate such a recovery in the reef fish assemblage. However, analyzing data from only the two sites that were visited in both 1999 and 2009 yielded qualitatively similar increases (the minimal degrees of freedom for both dates does not allow accurate tests of significances). Furthermore, we have been monitoring 45 reefs in the GOC on an annual basis for more than a decade [29], [41], [42], and have visited CPNP throughout the year since 2005 to study the behavior of large groupers (*Mycteroperca jordani*, *M. rocacea*) and characterize reef fish spawning aggregations. These observations allow us to confidently validate our results describing a fish community that rebounded remarkably from 1999 to 2009, from an area with few top predators similar to nearby open access areas to a no-take marine reserve dominated by top predators.

CPNP exhibited the largest absolute increase in biomass in a marine reserve reported in the literature [e.g.], [ 6], [ 43]–[45]. Previous studies have reported larger relative increases of biomass [see review in 4], but the magnitude of change was smaller. The most striking result is that full, complete recovery of a degraded fish community is possible (when placed in the right area and governed correctly), even to the level that is comparable to remote habitats that never have been impacted by fishing and other local human impacts [25], [46]. Such examples of “full” recovery are extremely rare [44], and we could not have expected that it occurred in only ten years.

The abundance of top predators and carnivores at CPNP is approaching the inverse trophic pyramid that characterizes reef fish assemblages that have faced little or no fishing pressure [25], [46]. The presence of sharks is another characteristic of healthy marine ecosystems [47], [48]. While not encountered on survey transects, large sharks (e.g. *Galeocerdo cuvier*, *Carcharhinus leucas*, *Triaenodon obesus*) were commonly observed at survey sites at CPNP but rarely or never observed at other reefs surveyed or at historic shark areas in the GOC [authors' pers. obs.; 33].

The ecological reasons for such a large increase in fish biomass probably include several factors: 1) the reserve was larger than the size of marine reserves studied by scientists (on average smaller than 10 km^2^) and thus can harbor permanent populations of large reef fishes with large home ranges, 2) the coral habitat was intact [38], 3) the reserve included spawning areas for large predators [49], and 4) it is located in an area of high productivity driven from both the spatial heterogeneity generated by long basaltic dykes that run parallel to the coast [50], and its location in the transition zone between the enclosed Gulf of California and the open waters of the Pacific Ocean.

The success of CPNP is greatly due to local leadership, effective self-enforcement by local stakeholders, and the general support of the broader community. Protected areas with locally managed resources and stakeholder buy-in can be more successful than areas with top down, federally mandated preservation [see 17]. This model is considered the most viable in rural settings where people rely on local natural resources for their livelihoods. Boat captains, dive masters, and local people in general participate in various activities to enforce the regulations of CPNP to visitors and among themselves, including surveillance, fauna protection (e.g. sea turtle nesting sites), and beach and ocean cleaning programs. These efforts have generated robust social bonds within the community [51], key elements for successfully managing aquatic resources and securing the livelihoods of the communities that depend on them [21].

The ecological successes of CPNP are steadily translating into economic benefits within the small (∼100 residents) rural village of Cabo Pulmo and the surrounding areas. A recent study found that the locally owned, small-scale tourism operators in Cabo Pulmo generated US$538,800 in 2006 and have continued to grow at a manageable rate [52]. This amount is generated by less than 30 people, working in five small businesses, and producing approximately US$18,000 per capita; an amount significantly higher than the per capita Gross National Income in Mexico. While tourism is not always the best option in ecologically sensitive areas (e.g., when excessive tourism demand for limited natural resources limits their availability for the local people and threatens ecosystem viability), these residents are showing ability for success, when local people use, manage, and benefit from their local resources.

## Materials and Methods

In 2009, we completed underwater visual surveys at 73 reefs in the GOC. Of those, 37 were the same sites surveyed by Sala et al. in 1999 [35] (including 2 in CPNP, Fig. 1A), and a total of 11 were located inside CPNP (Fig. 1B). In order to ensure the compatibility of data, we utilized the same survey methods described by Sala et al. [35]. Divers swam along 50 m transects observing and documenting fish species. Divers counted and estimated the size of all fishes belonging to all species within a five meter wide belt along each transect during two passes (250 m^2^ total). Different behavioral groups (mobile species versus territorial species), were surveyed during each pass to ensure that individuals were only counted once. At each site, we conducted four replicate transects at 20 m depth and four transects at 5 m depth. Using this method, we completed 435 total transects in 2009.

Typically, the majority of the area within any of Mexico's MPAs' boundaries allows for extractive activities, with only a small no-take area, known as “core zone,” designated for scientific research and monitoring. Seven of the ten MPAs in the GOC protect less than 6% of their total area through core zones (Table 1). In order to test CPNP's effectiveness as the only well enforced no-take marine reserve in the GOC, we divided our sites into three categories: (1) CPNP; (2) core zones in other MPAs, and (3) open access areas. For each category and for both survey periods (1999 and 2009), we calculated species richness, size structure, and biomass of all reef fishes. We also calculated biomass for each of four broad trophic groups: top predators, carnivores, zooplanktivores, and herbivores. We limited trophic categorization to these broad and robust groups because diets of species change ontogenetically and with the environment [53]. In order to maximize comparability with existing studies, biomass is expressed as tonnes per hectare. The biomass of individual fish was calculated using the allometric length-weight conversion: *W = a TL^b^*, where parameters *a* and *b* are species-specific constants, *TL* is total length in mm, and *W* is weight in grams. Length-weight fitting parameters were obtained from FishBase [54]. Differences in total transect biomass among trophic categories and between years were tested by means of a *t* test for unequal variances, and by a non-parametric Mann-Whitney *U*-test. Because fish schooling behaviour may also differ between years, we also tested for differences in relative biomass variance (the variance of the data standardized by the mean; an indicator of Poisson aggregation) using a variance-ratio *F*-test.

We also surveyed peer-reviewed scientific literature to compile a database of studies that document fish biomass values inside marine reserves and the surrounding fished areas to compare the changes in the reef fish community at CPNP to other marine reserves worldwide. We included only studies of fully-protected, no-take marine reserves and the nearby fished areas, and only those studies for which effects were measured for individual reserves in order to determine: (1) total fish biomass per unit area inside reserves; (2) ratio of fish biomass on reefs inside and outside reserves; and (3) annual rate of change of both total fish biomass and biomass in top trophic levels before and after reserve implementation. As it is a standard result in calculus that relative biomass increase can also be written as the rate of change of the logarithm of the variable [(1/*y*).d*y*/d*t* = d ln(*y*)/d*t*)], we estimated the annual relative rates of change between time zero and time *t* as ρ = ln(*x_t_*/*x*_0_)/*t*.

## Supporting Information

Table S1 **Analyses of variance of Simpsons diversity index obtained using the different species per each trophic group for every category.**
(PDF)
