# Supplementary material for: Large Recovery of Fish Biomass in a No-Take Marine Reserve
Source: PLoS One. 2011 Aug 12;6(8):e23601. doi: 10.1371/journal.pone.0023601 (PMC3155316; doi:10.1371/journal.pone.0023601)
Supplement: Table S1 — Analyses of variance of Simpsons diversity index obtained using the different species per each trophic group for every category. (PDF) [file pone.0023601.s001.pdf]

Table S1. Analyses of variance of Simpsons diversity index obtained using the different species per each trophic group for every category.

Categories	TL	1999	2009	F	P
CPNP	Top predators	1.09	1.52	5.77	<0.05
	Active predators	2.22	1.37	2.2	
	Benthic predators	3.85	3.70	0.12	
	Herbivores	2.22	2.22	0.00003	
	Zooplanktivores	1.28	1.14	1.34	
Core zones	Top predators	1.15	1.19	0.35	
	Active predators	1.30	1.52	0.09	
	Benthic predators	3.70	2.27	24.93	<0.0001
	Herbivores	2.78	2.04	6.91	
	Zooplanktivores	1.54	1.32	5.78	<0.05
Open access	Top predators	1.18	1.11	4.15	<0.05
	Active predators	1.47	1.47	0.05	
	Benthic predators	2.94	2.08	33.96	<0.0001
	Herbivores	2.22	1.89	8.43	<0.01
	Zooplanktivores	1.37	1.33	0.56	
